# Climbing and Medicine: Lessons from El Capitan for Medical Education

**DOI:** 10.1177/23821205251329668

**Published:** 2025-03-20

**Authors:** Paul B. Brandfonbrener

**Affiliations:** 1 University of California 8785 , San Francisco School of Medicine, San Francisco, CA, USA

**Keywords:** medical education, medical curriculum, medical students, learning theory, adult learning

## Abstract

In this reflective essay, the author explores the parallels between climbing El Capitan, a 3000-foot granite monolith in Yosemite Valley, and his medical education, with a focus on how Knowles’ andragogical model of adult learning shaped his approach to both. Drawing from his experience as an adult learner, he examines how the principles of self-directed learning, experiential learning, and motivation apply both to scaling a challenging rock face and to the demands of medical school. He reflects on how understanding the relevance of learning, taking ownership of progress, integrating previous experiences, and maintaining intrinsic motivation were key to success in both endeavors. Through this comparison, the author argues that Knowles’ model can offer valuable insights into improving medical education, particularly by creating curricula that are relevant, appropriately challenging, and grounded in real-world applications. This reflection underscores the importance of fostering a learning environment that is experiential, self-directed, and intrinsically motivating, enhancing both academic success and personal growth for medical students.

The portaledge swayed gently in the early morning June breeze, high above Yosemite Valley, as the wind streamed through the park and up the towering granite face of El Capitan. I was two days into a four-day climb of this 3000-foot monolith during summer break after my first year of medical school. Hanging 1000 feet above the valley, I woke to a pristine sunrise. In the moment, my thoughts were focused on the climb rather than my studies. But upon reflection, I realized the lessons I learned on the wall had parallels with my medical education.

I began climbing five years before attempting El Capitan, driven by the goal of scaling its immense face. As an adult learner, I faced challenges in learning this new skill—challenges that mirrored my experiences in medical school. After reflecting on both, I saw how Knowles’ andragogical model of learning, which outlines six key principles for adult education (Table 1),^1^ helped me succeed in climbing and could enhance my approach to learning medicine.

**Table 1.** The core principles of Knowles’ andragogical model of learning.

PRINCIPLE	DESCRIPTION
Need to know	Adult learners need to understand the relevance and importance of what they are learning to their lives. They need to know *why* they need to learn something.
Orientation to learning	Adults are often problem-centered in their learning. They want to apply what they learn to real-life situations.
Self-concept	Adults prefer to be self-directed in their learning. They want to take responsibility for their own learning process.
Prior experience	Adults bring a wealth of experience to the learning environment. This experience can be a valuable resource for learning.
Readiness to learn	Adults’ readiness to learn is often related to developmental tasks or life changes.
Motivation to learn	Adults are motivated to learn by internal factors such as job satisfaction, self-esteem, and quality of life.

## The Need to Know and Orientation to Learning

Two of Knowles’ assumptions are rooted in the idea that adults must understand why they need to learn something before they’re motivated to learn it. Furthermore, he asserts that adults are most motivated when they see how this new knowledge can solve real-life problems. In climbing, I had to acquire a wide range of skills—ropework, route finding, hauling gear—each of which contributed to the larger goal of summiting El Capitan. Understanding the practical application of each skill motivated me to learn. Additionally, I was only motivated to learn the specific techniques that would help me summit El Capitan. When I practiced skills that didn’t directly apply, my motivation waned.

In medicine, the same principles apply. Early in my medical training, I found that when professors began lectures by explaining how the material related to patient care, I became more engaged. When we were told how understanding ion gradients would allow us to appreciate the mechanism of diuretics that we would prescribe for our patients with heart failure, learning felt tangible. The more one can relate classroom learning to real life, the more engaging learning will be. Case-based learning or simulations, where students apply theory to real-world situations, is a way to accomplish this, and have been shown to improve learning and confidence in one's knowledge while reducing anxiety around novel clinical scenarios.^2^

## The Learners’ Self-Concept

According to Knowles, adults have a self-concept of being responsible for their own learning and decisions. As such, they have a psychological need to be treated as self-directed learners. When I was learning to climb, I took ownership of my training. I could choose which aspects of climbing I wanted to focus on—whether it was technical skills, strength training, or mental preparation—and I could shape my learning path to suit my needs. This autonomy was crucial for my progress, as it allowed me to take charge of my own development and learn in the way that felt most natural for me.

Similarly, medical students often benefit from some level of autonomy in their education. While it may not be feasible to craft entirely individualized curricula in a traditional medical school setting, giving students choices in how they engage with material can be beneficial. Offering content in various formats—written texts, video recordings, live lectures, or even novel hands-on experiences such as the use of virtual reality—can allow students to take a more active role in their learning. This flexibility helps foster a sense of self-direction, and the presentation of material through various forms has been shown to increase motivation and effectiveness in mastering complex material.^3^

## The Role of Learners’ Experiences

Knowles emphasizes the importance of incorporating learners’ previous experiences into the learning process. In climbing, I learned not only from my own experiences but also from the stories and advice of my climbing peers. Their insights—whether about gear, mental strategies, or technique—were invaluable in helping me avoid mistakes and improve my approach.

In medical education, peer learning plays a crucial role. Group discussions and collaborative learning allow students to share strategies for studying or handling clinical challenges. In my own training, group work helped me see different perspectives, which enriched my understanding of complex topics. Education research has similarly shown the efficacy of peer-assisted learning, with overall improvements in medical student performance, particularly in the acquisition of practical clinical skills.^4^

## Readiness to Learn

Adults are ready to learn when the material is relevant to their current life situation and capabilities, Knowles argues. When I first started climbing, I faced routes that were too difficult for my skill level. Trying to push past my abilities only led to frustration and risk. As I gradually took on progressively harder challenges, I built confidence and competence. This confidence allowed me to take on new challenges and solve novel problems that arose with my slowly developed fund of knowledge.

Similarly, in medicine, students are most ready to learn when the material is appropriately challenging. If the content is too advanced, students can become overwhelmed; if it's too basic, they lose engagement. This principle aligns with Vygotsky's concept of the “zone of proximal development,” where students learn best when the material is just beyond their current ability but still within reach with support.^5^ A well-designed curriculum should offer incremental challenges that align with students’ growing expertise. This approach acknowledges that the development of knowledge takes time, all the while allowing students to learn how to confidently apply their newly acquired skills to novel problems—a vital aspect to both a career in medicine, where no two patients are alike, and big wall climbing, where unforeseen challenges abound.

## Motivation

The final principle in Knowles’ model is intrinsic motivation. Adults are most motivated when they feel personally invested in the learning process. For me, climbing El Capitan provided a concrete, challenging goal. Every new skill I learned was a step toward that goal, which kept me focused and determined. My intrinsic motivation was key to overcoming obstacles and pushing through difficult moments.

In medicine, intrinsic motivation is equally powerful. As medical students, we often face intense challenges, and it's easy to lose motivation during difficult stretches. However, when students can connect their studies to their personal goals—whether it's a passion for patient care, a desire to contribute to medical research, or the aspiration to work in a specific specialty—it can be easier to persist through the hardships of medical training. Structured goal setting for medical students has been shown to increase accountability, self-reflection, and faculty engagement, contributing to overall student success and continued motivation.^6^ Encouraging students to set personal goals and reflect on their motivations for entering the field can increase their resilience and drive.

## The Summit

Two days later, I stood on top of El Capitan, bathed in the afternoon sun, sore and exhausted but elated. The climb had been grueling, but the satisfaction of reaching the top was immense. Reflecting on my climbing journey, I recognized how it paralleled my experiences in medical school. Both require immense personal responsibility, careful decision-making, and perseverance in the face of challenges.

Applying Knowles’ andragogical model to both climbing and medicine has shown me how important it is to structure learning in a way that is relevant, self-directed, experiential, appropriately challenging, and motivated by real-world applications. By fostering these elements, medical educators can create a more effective, engaging learning environment for students.

My experience also highlighted the value of drawing from life experiences outside the classroom. Climbing El Capitan may seem worlds apart from studying medicine, but the skills and lessons I learned on that rock face have made me a better learner—and, ultimately, a better future physician. Encouraging students to integrate their diverse experiences into their medical education can help them grow not only as clinicians but also as well-rounded individuals. In the end, both climbing and medicine are about problem-solving, resilience, and applying what we learn to real-world challenges. By embracing Knowles’ principles, we can foster more effective learning in medicine and whatever other fields bring us meaning.

## References

[1] Knowles MS Holton E Swanson RA . The Adult Learner: The Definitive Classic in Adult Education and Human Resource Development. 5th ed. Gulf Pub. Co., 1998.

[2] Yu JH Chang HJ Kim SS , et al. Effects of high-fidelity simulation education on medical students’ anxiety and confidence. PLoS One. 2021;16(5):e0251078. doi:10.1371/journal.pone.0251078. PMC8118241 33983983

[3] Stepan K Zeiger J Hanchik S , et al. Immersive virtual reality as a teaching tool for neuroanatomy. Int Forum Allergy Rhinol. 2017;7(10):1006-1013. 28719062 10.1002/alr.21986

[4] Brierley C Ellis L Reid ER . Peer-assisted learning in medical education: a systematic review and meta-analysis. Med Educ. 2022;56(4):365-373. doi:10.1111/medu.14672 34595769

[5] Vygotsky L . Mind in Society: The Development of Higher Psychological Processes. Harvard University Press; 1978.

[6] Larsen DP Naismith RT Margolis M . High-frequency learning goals: using self-regulated learning to influence day-to-day practice in clinical education. Teach Learn Med. 2017;29(1):93-100. doi:10.1080/10401334.2016.1230501 27813672

